# Supplementary material for: An Efficacious Transgenic Strategy for Triple Knockout of Xeno-Reactive Antigen Genes GGTA1, CMAH, and B4GALNT2 from Jeju Native Pigs
Source: Vaccines (Basel). 2022 Sep 8;10(9):1503. doi: 10.3390/vaccines10091503 (PMC9505423; doi:10.3390/vaccines10091503)

An efficacious transgenic strategy for triple knockout of xenoreactive antigen genes GGTA1, CMAH, and B4GALNT2 from Jeju Native Pigs

Seongwon Yoon, Seulgi Lee, Chungyu Park, Hyunyong Choi, Minwoo Yoo, Sang Chul Lee, Cheol-Ho Hyun, Nameun Kim, Taeyoung Kang, Eugene Son, Mrinmoy Ghosh, Young-Ok Son, Chang-Gi Hur

Original images for gels

Uncropped gel images related to Figure 2B

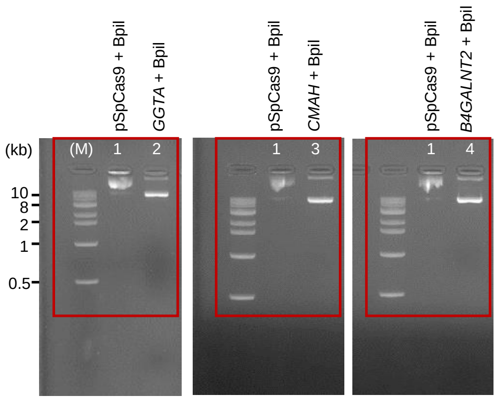

Uncropped gel images related to Figure 2C

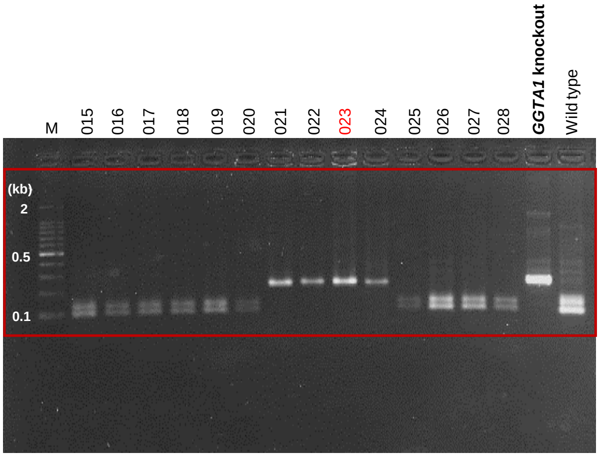

Uncropped gel images related to Figure 2D

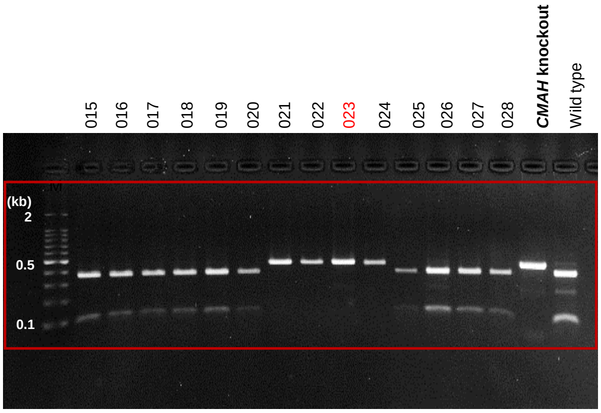

Uncropped gel images related to Figure 2E

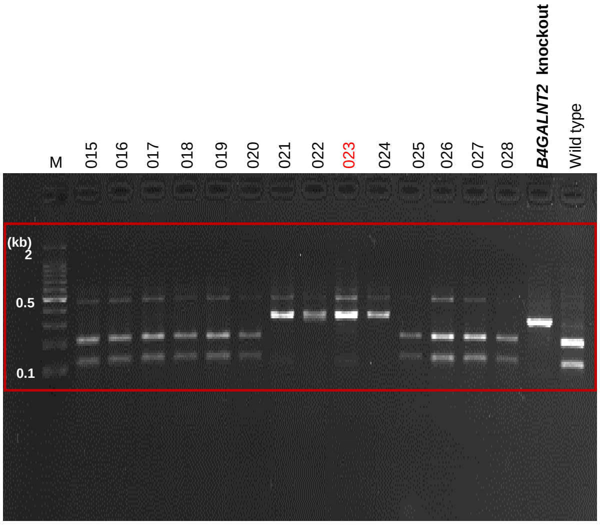

Supplement: Supplementary file 1 [file vaccines-10-01503-s001.zip › vaccines-1903560-Supplementary.pdf]
